# Identification of Genes Associated with Gut Microbiota Metabolites in Type 2 Diabetes Mellitus Complicated by Acute Cerebral Infarction Based on Bioinformatics and Mendelian Randomization

**DOI:** 10.3390/biomedicines14102371

**Published:** 2026-10-17

**Authors:** Guimei Zhang, Jiasheng Ju, Run Zhang, Xiuyuan Xu, Lifeng Li, Jing Zhao, Haibing Ju

**Affiliations:** 1 Department of Endocrinology, 920th Hospital of the Joint Logistics Support Force of the Chinese People’s Liberation Army, Kunming 650032, China; zgmhzxzyh@163.com (G.Z.); 18183891141@163.com (X.X.); zhaobo200610@163.com (L.L.); zhaojing-missing@163.com (J.Z.); 2 Department of Neurosurgery, Zhujiang Hospital of Southern Medical University, Guangzhou 510282, China; jnjiashen@163.com (J.J.) zhangrun2028@163.com (R.Z.)

**Keywords:** type 2 diabetes mellitus, acute cerebral infarction, gut microbiota, Mendelian randomization, *ANPEP*, *CYP1B1*

## Abstract

**Background:** Type 2 diabetes mellitus (T2DM) and acute cerebral infarction (ACI) are globally prevalent diseases that impose substantial public health burdens. While gut microbiota (GM) is increasingly recognized to regulate metabolic and immune homeostasis linked to both diseases, its shared genetically predicted microbial taxa and underlying metabolite–gene regulatory mechanisms in T2DM and ACI remain unclear, highlighting the need for systematic investigation. Accordingly, this study aimed to elucidate their regulatory mechanisms. **Methods:** Transcriptomic datasets, genome-wide association study (GWAS) data, and GM data were retrieved from public databases. Key microbial taxa were identified using two-sample Mendelian randomization (MR) and 16S rRNA sequencing. Metabolite–target genes (MTGs) were predicted via public databases. Key metabolites were screened based on Lipinski’s rules and low toxicity, and key genes were prioritized by molecular docking. Regulatory networks and potential drugs were predicted using public databases. **Results:** Using the FinnGen diabetes endpoint as a proxy for T2DM-related genetic susceptibility, MR analysis identified 12 candidate microbial taxa shared by both diseases. Further intersection with the top 30 microbial taxa from 16S rRNA sequencing confirmed two key microbial taxa (*Alistipes* and *Bifidobacterium*). Subsequently, 602 MTGs were identified for metabolites derived from the key microbial taxa. After druggability evaluation and toxicity profiling, nine key metabolites with favorable druggability and low toxicity were identified. Molecular docking predicted binding affinities between these key metabolites and the encoded proteins, suggesting *ANPEP* and *CYP1B1* as candidate key genes. Additionally, *ANPEP* interacted with 12 transcription factors (TFs), while *CYP1B1* was regulated by six TFs and 13 microRNAs that further interacted with 26 long noncoding RNAs. Sixty-eight and 32 potential drugs targeting *ANPEP* and *CYP1B1*, respectively, were identified, providing candidates for translational research. **Conclusion:** This study systematically identified *ANPEP* and *CYP1B1* as candidate key genes in the GM-associated regulatory network underlying T2DM-related genetic susceptibility and ACI, providing candidate molecular targets and candidate compounds for the development of GM-based targeted therapies.

## 1. Introduction

The prevalence of diabetes mellitus (DM) continues to rise globally. According to data from the International Diabetes Federation, the total number of individuals living with DM aged 20–79 in China reached 148 million in 2024 [1]. Over 90% of these cases correspond to type 2 diabetes mellitus (T2DM). Cardiovascular and cerebrovascular complications are among the leading causes of mortality in patients with T2DM. T2DM serves as an independent risk factor for acute cerebral infarction (ACI) [2]. ACI accounts for approximately 69.6% of incident stroke cases in China [3] and represents one of the leading contributors to mortality and disability globally [4]. The coexistence of the two disorders not only severely impairs patients’ quality of life but also imposes a substantial burden on healthcare systems. Early screening, timely diagnosis, and clinical intervention are critical for alleviating neurological deficits and reducing the risk of sequelae in patients with T2DM complicated by ACI. Accordingly, accumulating studies have focused on identifying novel therapeutic targets and diagnostic biomarkers for T2DM complicated by ACI.

In recent years, a growing body of research has centered on the regulatory effects of gut microbiota (GM) and its metabolites in T2DM and ACI. Kristensen et al. [5] reported that GM homeostasis was disrupted before stroke onset, indicating that tracking characteristic GM shifts may facilitate early prediction and prevention of ischemic stroke. Wang et al. [6] verified a causal association between GM dysbiosis in T2DM patients and post-stroke brain injury: after cerebral infarction, T2DM-related GM dysbiosis further impaired the intestinal mucosal barrier, enabling lipopolysaccharides to enter systemic circulation, compromise the blood–brain barrier, and aggravate cerebral injury. The same group further validated this mechanism via animal experiments, demonstrating that fecal microbiota transplantation from T2DM mice with improved diabetic phenotypes reduced infarct volume in recipient animals. These findings suggest that the GM–metabolite axis may contribute to the onset and progression of T2DM complicated by ACI, and may represent a potential therapeutic target for this comorbidity. However, the mechanisms underlying this regulatory process remain poorly characterized and warrant further investigation.

Transcriptomics has broad applications in clinical diagnosis and drug development. Given that the onset and progression of T2DM and ACI are closely linked to altered gene expression, transcriptomic profiling is particularly well-suited to dissecting the mechanisms underlying both conditions. Mendelian randomization (MR) leverages single-nucleotide polymorphisms (SNPs) as instrumental variables to infer causal relationships between exposures and outcomes [7]. MR studies have suggested genetically predicted associations between specific gut microbial taxa and ischemic stroke risk [8]. Nevertheless, systematic research integrating transcriptomic and MR analyses remains limited for characterizing the mechanisms through which the GM and its metabolites mediate the development of T2DM complicated by ACI.

This study focused on the molecular mechanisms and potential intervention strategies for T2DM complicated by ACI. We first integrated public databases, MR analysis, and 16S rRNA sequencing to identify key gut microbial taxa. Differential expression analysis and gut microbial metabolite–target prediction were then combined to identify shared genes and associated pathways. Finally, drug-likeness and toxicity assessments and molecular docking were used to prioritize key genes, followed by construction of a multidimensional “GM–metabolite–target–pathway” network and prediction of upstream regulators and candidate drugs (Figure 1).

## 2. Materials and Methods

### 2.1. Data Sources and Processing

Two microarray datasets, GSE20966 (platform GPL1352) and GSE16561 (platform GPL6883), were retrieved from the NCBI Gene Expression Omnibus (GEO, https://www.ncbi.nlm.nih.gov/geo/ accessed on 13 October 2025). GSE20966 comprises pancreatic β-cell specimens from 10 T2DM patients and 10 healthy controls [9]. GSE16561 covers whole blood samples collected from 39 ACI patients and 24 matched healthy volunteers [10].

GWAS summary statistics for T2DM and ACI were obtained from the IEU OpenGWAS database (https://gwas.mrcieu.ac.uk/ accessed on 14 October 2025). For T2DM, the “finn-b-E4_DIABETES” dataset was selected using “Diabetes mellitus” as the keyword (total sample size: 218,792; control sample size: 183,185; number of disease samples: 35,607; number of SNPs: 16,380,466; ethnic group: European). The FinnGen endpoint finn-b-E4_DIABETES includes type 1, type 2 and unspecified diabetes. It was used as a proxy for adult diabetes-related genetic susceptibility, not as a strictly T2DM-specific endpoint. For ACI, the “ukb-d-I63” dataset was chosen with “Cerebral infarction” as the keyword (total sample size: 361,194; control sample size: 358,841; number of disease samples: 2353; number of SNPs: 10,889,323; ethnic group: European). Additionally, GWAS data related to GM were acquired from the MiBioGen database (https://www.mibiogen.org/ accessed on 14 October 2025) [11].

Although the transcriptomic datasets differ in tissue source and the GWAS and 16S cohorts differ in ancestry, they were integrated to generate hypotheses regarding a systemic gut microbiota–metabolite–gene axis rather than to establish direct tissue-specific causality. Tissue source, ancestry and cohort heterogeneity were therefore considered in the interpretation of all downstream findings.

### 2.2. Two-Sample MR Analysis

To explore potential genetically predicted associations GM and the two outcomes (T2DM and ACI), two-sample MR analysis was performed with GM as the exposure and each disease as the outcome. The following MR analysis was primarily conducted using the “TwoSampleMR” package (v 0.6.8) [12].

The “extract_instruments” function was employed to input exposure factors, screen IVs, and identify effective IVs. IVs significantly correlated with exposure factors were selected based on a threshold of *p* < 5 × 10^−6^; subsequently, SNPs in linkage disequilibrium (LD) were pruned (clump = TRUE, window size = 10 kb, nSNPs ≥ 3 and r^2^ = 0.001). Using T2DM GWAS data and the selected IVs, the study removed IVs that were significantly correlated with the outcome (proxies = TRUE; rsq = 0.8). In addition, the F-statistic was computed using the formula to assess the strength of the instrumental variables and reduce potential weak-instrument bias. SNPs with *F*-statistics ≤ 10 were excluded.

F = (N − k − 1) R^2^/[k (1 − R^2^)] (1)

To investigate the causal relationships between GM and T2DM and ACI, respectively, the “harmonise_data” function was used to standardize effect alleles and effect sizes. In addition, inverse variance weighting (IVW) was adopted as the primary method, supplemented by the weighted median method, MR-Egger regression, the simple mode, and the weighted mode to conduct MR analyses of GM, T2DM, and ACI. For the MR association screening, a nominal *p* < 0.05 was considered statistically significant. No false discovery rate (FDR) or Bonferroni correction was applied at this exploratory screening step; therefore, these associations were used only to retain candidate microbial taxa for subsequent intersection, random forest and sensitivity analyses. Among candidate exposures, those with an OR > 1 were defined as risk factors, while those with an OR < 1 were considered protective factors. Analytical results were visualized using the “mr_scatter_plot,” “mr_forest_plot,” and “mr_funnel_plot” functions to generate scatter plots, forest plots, and funnel plots, respectively.

Heterogeneity analysis was performed using the “mr_heterogeneity” function. MR analyses were performed using the random-effects IVW model if heterogeneity was present (Q_*p* < 0.05), or the fixed-effects IVW model if no heterogeneity was detected (Q_*p* > 0.05). Subsequently, a horizontal pleiotropy test was performed through the “mr_pleiotropy_test” function (*p* > 0.05). Moreover, the “mr_leaveoneout” function was employed to perform the leave-one-out sensitivity test, and the “mr_leaveoneout_plot” function was used to generate the corresponding plot. Specifically, each SNP was sequentially removed, and the effect of the remaining SNPs on the outcome was evaluated. If the MR results changed significantly after excluding a specific SNP, it indicated that the results were sensitive to the removal of this SNP; therefore, the SNP was excluded before re-performing the analysis.

To assess the directionality of the inferred associations, the screened causal gut microbial taxa were set as exposures, with T2DM and ACI as outcomes. Steiger directionality tests were performed using the “steiger_filtering” function. A test output of TRUE with *p* < 0.05 was considered to support the hypothesized direction from exposure to outcome. Finally, the intersection of microbial taxa causally associated with T2DM and those associated with ACI was extracted to identify shared causal gut microbial taxa underlying T2DM complicated by ACI.

### 2.3. Sample Collection and Sequencing

All participants were recruited from the 920th Hospital of the Joint Logistics Support Force of the Chinese People’s Liberation Army (hereinafter referred to as “the 920th Hospital”). Prior to sample collection, participants who had taken antibiotics, probiotics, or prebiotics within the previous 3 months, those receiving long-term oral antiplatelet therapy, and patients with type 2 diabetes mellitus who used metformin or acarbose in the preceding 3 months were excluded. Data on other microbiota-altering medications (laxatives, iron supplements, antidepressants, anxiolytics, antihistamines) were not collected, representing potential residual confounders. Diagnoses of T2DM and ACI were confirmed according to established clinical guidelines [13,14]. Written informed consent was obtained from all participants. This study strictly adhered to the Declaration of Helsinki and was approved by the Medical Ethics Committee of the 920th Hospital prior to its commencement (No: 2025-057-01). A total of 40 participants were enrolled, including 20 patients with ACI and 20 patients with T2DM complicated by ACI. Fecal samples were collected upon initial admission. All participants were Han Chinese from the Kunming region, China. This single-center, same-region design reduces regional and ethnic heterogeneity, and dietary patterns may be relatively similar; however, dietary intake was not systematically recorded using a standardized questionnaire, which remains a limitation.

All fecal samples were stored at −80 °C and transported on dry ice to the testing facility for 16S rRNA sequencing. Sequencing was performed on the Illumina NovaSeq 6000 platform (Illumina Inc., San Diego, CA, USA). After the data were downloaded and primer sequences were removed using Cutadapt(v5.0), quality control—including quality filtering, denoising, assembly, and chimera removal—was performed using the DADA2 algorithm within QIIME2 (v2020.11). This process generated representative sequences and amplicon sequence variant (ASV) abundance tables. The representative sequences were then taxonomically annotated by alignment against the SILVA database (v138) using the q2-feature-classifier plugin with default parameters. The 16S cohort was designed as an exploratory comparison between ACI and T2DM-ACI; healthy and T2DM-only controls were not included. Residual confounding by diet, medications, hospitalization and vascular risk factors cannot be fully excluded.

Alpha and beta diversity analyses were performed using QIIME2. Alpha diversity was assessed using the Chao1 and Shannon indices. Beta diversity was calculated using unweighted UniFrac distances and visualized by principal coordinate analysis (PCoA). Statistical differences in beta diversity between groups were assessed using permutational multivariate analysis of variance (PERMANOVA). Linear discriminant analysis effect size (LEfSe) was conducted to detect differentially abundant taxa at five taxonomic ranks (phylum, class, order, family, genus). Taxa with an LDA score > 2 and *p* < 0.05 were regarded as significantly enriched. Random forest (RF) analysis was carried out using the “randomForest” package in R to screen key gut bacterial genera responsible for intergroup differences. Variable importance plots were generated to rank the discriminatory power of each genus.

### 2.4. Identification of Key Microbial Communities

The VennDiagram package (v1.7.3) [15] was used to calculate the overlap between candidate gut microbial taxa from MR analysis and the top 30 taxa screened by random forest, and a Venn diagram was generated accordingly. Taxa within this overlapping set were defined as key gut microbial taxa.

### 2.5. Identification of Candidate Genes

First, key GM taxa and their corresponding metabolite data were retrieved from the gutMGene database (http://bio-computing.hrbmu.edu.cn/gutmgene/ accessed on 22 October 2025 Table S1). Then, the simplified molecular-input line-entry system (SMILES) structures of all metabolites were queried from the Public Chemical Database (Pubchem) (https://pubchem.ncbi.nlm.nih.gov/ accessed on 22 October 2025). Next, the SMILES structures of all metabolites were submitted to the Similarity Ensemble Approach (SEA, https://sea.bkslab.org/ accessed on 22 October 2025) and SwissTargetPrediction (STP, http://www.swisstargetprediction.ch/ accessed on 22 October 2025) platforms for target prediction. Finally, the overlapping genes identified by both platforms were designated as metabolite–target genes (MTGs) (Table S1).

To identify differentially expressed genes (DEGs) associated with T2DM and ACI, we performed differential expression analysis using the “limma” package (v3.56.2) [16] on the GSE20966 and GSE16561 datasets, with comparisons between disease and control groups conducted for each dataset. Differential expression was defined as *p* < 0.05 and |log_2_FC| > 0.5 without additional BH-FDR correction; these DEGs were treated as exploratory candidates for subsequent intersection. The “ggplot2” (v3.4.1) [17] and “ComplexHeatmap” (v2.14.0) [18] packages were used to generate volcano plots and heatmaps for DEG visualization, respectively. Using the DEGs identified above, we plotted Venn diagrams with the “VennDiagram” package to identify genes that were consistently upregulated or downregulated in both the T2DM and ACI datasets. These consistently dysregulated genes were retained as comorbidity-associated DEGs. The intersection of these comorbidity-associated DEGs and the MTGs was then determined to identify candidate comorbidity genes.

To investigate the molecular functions of candidate genes and the key pathways in which they are involved, we performed Gene Ontology (GO) and Kyoto Encyclopedia of Genes and Genomes (KEGG) enrichment analyses using the “clusterProfiler” package (v4.2.2) [19]. A threshold of *p*.adjust< 0.05 was adopted to define significantly enriched terms. GO analysis covered three primary categories: biological process (BP), molecular function (MF), and cellular component (CC). The top 10 most significantly enriched terms from each category were visualized using the “ggplot2” package.

### 2.6. Screening and Validation of Key Genes

The drug likeness and toxicity of metabolites interacting with candidate genes were evaluated using the SwissADME (http://www.swissadme.ch/ accessed on 30 October 2025) and ADMETlab (https://admetmesh.scbdd.com/ accessed on 30 October 2025) platforms. Drug likeness was assessed based on Lipinski’s rules: (1) number of Lipinski’s rule violations ≤ 1; (2) molar octanol-water partition coefficient (MlogP) ≤ 4.15; (3) number of hydrogen bond acceptors (HBA) ≤ 10; (4) number of hydrogen bond donors (HBD) ≤ 5; and (5) topological polar surface area (TPSA) < 140. Subsequently, metabolites complying with Lipinski’s Rule of Five were subjected to further ADMET property analysis via ADMETlab 2.0. Multiple toxic endpoints of these metabolites were detected, including hERG (human Ether-à-go-go-Related Gene) potassium channel blocking activity, human hepatotoxicity, respiratory toxicity, drug-induced liver injury (DILI), rat oral acute toxicity, carcinogenicity, skin sensitization, and median lethal concentration (LC_50_DM). Key metabolites were considered nontoxic or of low toxicity if they satisfied the following criteria: a negative result for LC_50_DM (defined as LC_50_DM > 1) and negative results for the other seven indicators (each defined as ≤0.5). Metabolites screened through the above two platforms were designated as core metabolites.

In addition, the GM and target genes corresponding to core metabolites were extracted from Table S1 in this study, and the intersection of target genes and candidate genes was taken to obtain core genes. To explore the interactions among GM, core genes, core metabolites, and all KEGG signaling pathways, Cytoscape (v3.10.2) [20] was used to construct a network diagram of their relationships.

Finally, to investigate the binding affinity between these core metabolites and the proteins encoded by the core genes, metabolite structures were downloaded from the PubChem database (https://pubchem.ncbi.nlm.nih.gov/ accessed on 6 November 2025), and protein structures (encoded by the core genes) were retrieved from the Research Collaboratory for Structural Bioinformatics Protein Data Bank (RCSB PDB) database (https://www.rcsb.org/ accessed on 6 November 2025). Molecular docking between these key metabolites and the proteins encoded by the core genes was performed using CB-Dock2 (https://cadd.labshare.cn/cb-dock2/php/index.php accessed on 6 November 2025). In general, a binding energy < −4.25 kcal/mol (more negative values indicate stronger binding) was considered to indicate favorable binding affinity between the proteins encoded by the core genes and their corresponding metabolites. PyMOL(v2.2.0) was used to visualize the complex in three dimensions. Among the core genes, those whose binding energy with key metabolites met the preset criteria were selected and prioritized as candidate key genes.

These analyses are hypothesis-generating and cannot establish target engagement, interaction direction or biological function.

### 2.7. Regulatory Networks of Key Genes and Drug Prediction Analysis

First, microRNAs (miRNAs) interacting with key genes were jointly predicted via three independent databases: miRTarBase (http://mirtarbase.cuhk.edu.cn/ accessed on 13 November 2025), miRDB (http://mirdb.org/ accessed on 13 November 2025), and TargetScan (http://www.targetscan.org/ accessed on 13 November 2025). Only miRNAs predicted across all three databases were retained as candidate miRNAs. Subsequently, long noncoding RNAs (lncRNAs) targeting these candidate miRNAs were predicted using the StarBase database (http://starbase.sysu.edu.cn/ accessed on 13 November 2025), and only lncRNAs satisfying the criterion of “pancancerNum” > 6 were preserved. Regulatory networks reflecting TF–messenger RNA (mRNA) and mRNA–miRNA–lncRNA crosstalk were initially constructed in Cytoscape (v3.10.2). To further elucidate the molecular regulatory mechanisms of key genes, transcription factors (TFs) targeting these genes were screened from the ChIPBase database (http://rna.sysu.edu.cn/chipbase/ accessed on 14 November 2025). Key TFs for each key gene were screened based on the criterion that the sum of upstream and downstream detected sample numbers exceeded 12. To investigate the TF binding sites (TFBS) of key genes, the promoter regions of the genes (herein defined as the sequence from 2000 bp upstream to 100 bp downstream of the gene start site) were retrieved using the National Center for Biotechnology Information (NCBI) database (https://www.ncbi.nlm.nih.gov/ accessed on 14 November 2025). Subsequently, TFBS were predicted with the hTFtarget database (https://guolab.wchscu.cn/hTFtarget#!/ accessed on 15 November 2025), and the regulatory TFs that bind to these sites were screened with the criterion of Q_value < 0.01. Cytoscape (v3.10.2) was adopted to visualize the TFBS–key gene regulatory network.

In addition, the GeneMANIA database (https://genemania.org/ accessed on 15 November 2025) was used to identify genes with functions similar to those of the key genes (Species = Homo sapiens). Co-expression networks were then constructed between the key genes and the top 20 functionally similar genes to characterize their associations.

To delve into the interrelationships between key genes and potential drugs, the Drug–Gene Interaction Database (DGIdb, https://beta.dgidb.org/ accessed on 15 November 2025) was used to investigate the potential drugs associated with key genes, and a drug–key gene interaction network was constructed using Cytoscape (v3.10.2).

### 2.8. Statistical Analysis

Bioinformatics analyses were performed using R (v4.3.3). Differences between two groups were assessed using the Wilcoxon rank-sum test. A two-sided *p* < 0.05 was considered statistically significant.

## 3. Results

### 3.1. Candidate Microbial Communities Genetically Predicted to Be Associated with T2DM and ACI

A total of 1417 and 2118 SNPs were selected as instrumental variables for MR analyses of GM with T2DM and ACI, respectively (Table S2). The F-statistics of all selected IVs exceeded 10.

IVW analysis revealed that 34 and 24 GMs exhibited genetically predicted associations with ACI and T2DM, respectively, with small effect sizes (*p* < 0.05) (Table S3); these taxa should be regarded as candidate microbial features identified through genetic prediction rather than definitive causal factors. Among these, 26 microbial taxa were identified as protective factors against ACI (OR < 1), including *Bifidobacterium* (OR = 0.999, 95% CI = 0.998–0.999, *p* = 1.83 × 10^−5^), and eight were identified as risk factors (OR > 1), such as *Alistipes* (OR = 1.006, 95% CI = 1.002–1.009, *p* = 0.001). Because the FinnGen diabetes endpoint includes multiple diabetes subtypes, the T2DM associations reported below should be interpreted as reflecting T2DM-related genetic susceptibility rather than strict T2DM-specific causality. For T2DM, 15 microbial taxa with protective associations, such as *Alistipes* (OR = 0.997, 95% CI = 0.994–0.999, *p* = 0.019) and nine risk-associated microbial taxa, including *Bifidobacterium* (OR = 1.001, 95% CI = 1.000–1.001, *p* = 0.042) were screened. The scatter plots for all 58 GMs showed that the MR-Egger intercepts were close to 0, indicating no significant horizontal pleiotropy (Figure S1), and the forest plots further corroborated these causal associations (Figure S2). The funnel plots exhibited a generally symmetric distribution, consistent with the assumption of independent assortment (Figure S3). To assess the robustness of the MR findings, we conducted sensitivity analyses. Cochran’s Q test revealed no significant heterogeneity among the SNPs for any of the 58 GMs (*p* > 0.05) (Table S4); therefore, the fixed-effects IVW model was applied. MR-Egger regression intercepts did not deviate significantly from zero, suggesting no evidence of horizontal pleiotropy (Table S5). Leave-one-out analysis showed that the causal estimates were not driven by any single SNP, confirming the stability of the results (Figure S4). Steiger directionality tests supported the hypothesized direction from exposure to outcome for all 58 GMs (Table S6), supporting that the inferred effects proceeded from GM exposure to disease outcome rather than the reverse. Collectively, these analyses identified 34 and 24 GMs with robust genetically predicted associations with ACI and T2DM, respectively.

Intersecting the 34 GMs associated with ACI and the 24 GMs associated with T2DM yielded 12 shared candidate microbial taxa for subsequent analyses (Figure 2A).

### 3.2. Gut Microbiome Characteristics of Patients with T2DM-ACI

There was no significant difference in alpha diversity between the ACI and T2DM-ACI groups (*p* > 0.05) Figure S5). In contrast, beta diversity analysis exhibited distinct microbial community separation between the two groups, and PERMANOVA verified significant structural differences in gut microbial composition (R^2^ = 0.039, *p* = 0.016) (Figure 2B).

Community structure profiling combined with LEfSe analysis identified differentially abundant genera between the two groups. At the genus level, 10 taxa were significantly enriched in the ACI group, including *Muribaculaceae_g_Unclassified*, *Eubacterium_eligens_group*, *Haemophilus*, *Lachnospiraceae_UCG-003*, *Rikenella*, and *Proteus*. In contrast, *Eisenbergiella*, *Slackia*, *Turicibacter*, and *Coprobacillus* were enriched in the T2DM-ACI group (Figure 2C–F).

Random forest analysis was subsequently performed to screen discriminatory bacterial genera between the two groups. The results indicated that *Bifidobacterium* exhibited the highest variable importance score and was the most discriminatory taxon separating the two cohorts in the model (Figure 2G).

### 3.3. Identification of Key Microbial Communities

The intersection of the 12 candidate GMs from the MR analysis and the top 30 genera identified by random forest analysis yielded two shared taxa: *Alistipes* and *Bifidobacterium*. These were designated as key gut microbial taxa (Figure 2H).

### 3.4. Screening and Analysis of Candidate Genes Associated with T2DM and ACI

Based on the abovementioned analyses, we predicted a total of 43 key microbiota-related metabolites (Table S7) and 602 MTGs (Table S1) using the SEA and STP databases.

Differential expression analysis identified 676 significant DEGs in the GSE16561 dataset. Among these, 507 were upregulated and 169 were downregulated in ACI samples relative to normal controls. In the GSE20966 dataset, 1061 DEGs were identified, of which 703 were upregulated and 358 were downregulated in T2DM samples relative to normal controls. The top 10 upregulated and top 10 downregulated genes were visualized by volcano plots and heatmaps, respectively (Figure 3A–D, Table S8). Venn diagram analysis further identified 23 upregulated and 4 downregulated comorbidity-associated DEGs (Figure 3E). The intersection of these 27 comorbidity-associated DEGs with the 602 MTGs yielded 4 candidate comorbidity genes: *ANPEP, CTSS, CYP1B1*, and *DYRK2* (Figure 3F).

Functional enrichment analyses were conducted to characterize the biological functions and pathway associations of the candidate genes. A total of 169 significantly enriched GO terms were identified after BH-FDR correction (*P.*adjust < 0.05), including 140 BPs, 3 CC, and 26 MFs (Table S9). Specifically, “trabecular meshwork development” was the top enriched term in BPs, while “endolysosome lumen” and “steroid hydroxylase activity” ranked first in CC and MFs, respectively (Figure 3G). For pathway enrichment, nine KEGG pathways were significantly enriched after BH-FDR correction (*P.*adjust < 0.05), such as “Phagocytosiss”, “Tryptophan metabolism”, and “Steroid hormone biosynthesis” (Figure 3H and Table S9). These enrichment results indicated that the candidate genes were primarily involved in processes such as phagocytosis and antigen presentation, tryptophan and steroid hormone metabolism, the renin–angiotensin–aldosterone system (RAAS), cytochrome P450-mediated xenobiotic metabolism, and glutathione metabolism, thereby providing potential insights into the mechanisms underlying the comorbidity of T2DM and ACI.

### 3.5. Screening and Validation of Key Genes

Drug-likeness assessment of the 63 metabolites associated with the four candidate genes identified 54 that fully complied with Lipinski’s rule of five (Table S10). Subsequent ADMET profiling of these 54 metabolites identified nine with no or low toxicity, which were designated as core metabolites (Table S10). Subsequently, the 18 GMs and 359 target genes corresponding to key metabolites were extracted from Table S1 in this study. Further overlap analysis of 359 target genes corresponding to key metabolites and four candidate genes yielded three core genes, namely *ANPEP, CTSS*, and *CYP1B1* (Table 1). In the regulatory network comprising three core genes, 18 GMs, nine key metabolites, and nine KEGG pathways, except for 3-Hydroxy-4-methoxybenzenepropanoic acid (HMCA), *ANPEP* interacted with the other eight key metabolites; these metabolites could interact with GMs such as *Bifidobacterium* and *Ruminococcus*, and *ANPEP* was associated with the “Renin–angiotensin system” and “Glutathione metabolism” pathways (Figure 4A).

Molecular docking analysis was conducted to explore the binding interactions between core genes and gut microbial metabolites. The protein encoded by *ANPEP* showed predicted favorable binding affinities with five metabolites, including 3- (3-hydroxyphenyl)propanoic acid, 3-phenylpropionic acid, citrulline, D-methionine, and methionine. The *CYP1B1* protein showed predicted favorable binding activity with HMCA. In contrast, the *CTSS* protein displayed relatively weak binding affinity for ethyl phenyllactate (Figure 4B–G, Table 2). Therefore, *ANPEP* and *CYP1B1* were prioritized as candidate core genes potentially associated with the comorbidity of T2DM and ACI.

### 3.6. Regulatory Networks of Key Genes and Drug Prediction Analysis

Regulatory prediction analysis was performed to screen the upstream TFs, miRNAs, and lncRNAs of the key genes. *CYP1B1* was predicted to interact with 13 miRNAs and 26 lncRNAs. In contrast, no miRNAs or lncRNAs were predicted to interact with *ANPEP* (Figure 5A). The constructed lncRNA–miRNA–mRNA regulatory network revealed extensive molecular interactions. For instance, multiple lncRNAs, including LINC02693, TOB1-AS1, and LINC02381, could modulate *CYP1B1* expression by targeting hsa-miR-27b-3p. Subsequently, the interaction network between key genes and TFs revealed that *ANPEP* interacted with 12 TFs, whereas *CYP1B1* interacted with 6 TFs. Five TFs, including SPI1, STAT3, CEBPB, ESR1, and FOXA1, were identified as common upstream regulators of both genes (Figure 5B). Corresponding TFBS could be predicted in the promoter region of *ANPEP*, with 170 TFs capable of binding to these sites; in contrast, 201 TFs bind to the TFBS within the promoter region of *CYP1B1* (Figure 5C). This suggested that they might exert regulatory effects on disease initiation and progression by targeting these key genes, providing novel insights into the transcriptional regulatory mechanisms underlying disease pathogenesis.

Additionally, the top 20 functionally similar genes (*ENPEP*, *EPHX2*, *MAF*, etc.) were obtained, and these genes were closely associated with “aminopeptidase activity”, “exopeptidase activity” and “metallopeptidase activity” (Figure 5D), which provides molecular-level insights into the functional network of the key genes and other critical physiological and pathological processes they are involved in, and facilitates further exploration of their mechanism of action in disease.

Furthermore, to explore potential targeted therapeutic drugs for the two key genes (*ANPEP* and *CYP1B1*), a drug-target matching analysis was performed, and the results identified 68 drugs that could target *ANPEP* (such as Chrysin) and 32 drugs that could target *CYP1B1* (such as Colchicine), respectively (Figure 5E). These results not only provided direct candidate drugs for targeted therapy but also offered theoretical support for elucidating the interplay between key gene targets and therapeutic agents, laying a foundation for subsequent translational research.

## 4. Discussion

The prevalence of T2DM and ACI continues to rise annually, with an increasingly younger onset age, constituting a serious global public health problem [21]. T2DM serves as a critical independent risk factor for stroke. Epidemiological evidence indicates that Chinese patients with diabetes exhibit a two-fold higher risk of ACI compared with non-diabetic individuals [22]. Patients with ACI complicated by T2DM typically present poorer clinical prognoses, including higher rates of disease recurrence, rehospitalization, disability, and mortality [2]. Notably, although rigorous glycemic control effectively reduces microvascular complications in T2DM patients, it fails to substantially lower the risk of stroke [23]. Therefore, identifying reliable early diagnostic biomarkers, elucidating the underlying molecular mechanisms, and exploring targeted intervention strategies for T2DM complicated by ACI are urgently required. Accumulating evidence has demonstrated that gut microbiota and its metabolites participate in the initiation and progression of both T2DM and ACI, representing promising therapeutic targets for preventing and treating T2DM and its associated cerebrovascular complications. Nevertheless, the genetically predicted links connecting gut microbial metabolites to T2DM and ACI remain poorly characterized and require experimental validation. In the present study, we integrated random forest screening and Mendelian randomization analyses to identify *Bifidobacterium* and *Alistipes* as key microbial genera. By further integrating transcriptomic data with gut microbiota metabolite–target gene predictions, followed by molecular docking validation, we identified *ANPEP* and *CYP1B1* as candidate key genes potentially linked to gut microbial metabolites closely linked to gut microbial metabolites in the context of T2DM complicated by ACI.

*CYP1B1* encodes a widely expressed monooxygenase implicated in metabolism, inflammation, angiogenesis, and anticancer drug resistance [24]. Previous studies have established a close association between *CYP1B1* expression and both T2DM and ACI. *CYP1B1* has been predicted to be a central target through which active components of sage regulate key pathways involved in T2DM pathogenesis and complications, including insulin resistance, advanced glycation end products and their receptors, and the aryl hydrocarbon receptor (AHR). This suggests that aberrant *CYP1B1* upregulation may contribute to T2DM pathogenesis by modulating these pathological pathways [25,26]. *CYP1B1* has also been shown to promote reactive oxygen species (ROS) production and activate the NF-κB pathway, thereby triggering inflammatory cascades that contribute to the onset and progression of atherosclerosis (AS) and T2DM [27]. Estradiol has been shown to significantly ameliorate peripheral immunosuppression and reduce infarct volume in female mouse models of stroke [28]. *CYP1B1* may regulate post-stroke estradiol levels by mediating its metabolic degradation [29]. In terms of signaling pathways, our results indicated that *CYP1B1* was associated with Tryptophan metabolism, Steroid hormone biosynthesis, and Metabolism of xenobiotics by cytochrome P450. Previous studies have identified *CYP1B1* as a key gene involved in cortisol-related biological processes, which may regulate stroke onset and progression through the Steroid hormone biosynthesis, and Metabolism of xenobiotics by cytochrome P450 pathways [30]. Our results are consistent with these findings, suggesting that *CYP1B1* may modulate cortisol responses through these pathways, thereby influencing the progression of T2DM complicated by ACI. Chen et al. [31] demonstrated that elevated *CYP1B1* expression promotes tryptophan catabolism through the kynurenine pathway by upregulating tryptophan 2,3-dioxygenase 2, while concurrently activating monocytes to release pro-inflammatory cytokines, thereby amplifying the inflammatory cascade. Furthermore, molecular docking revealed that HMCA, a metabolite produced by *Eubacterium ramulus* and *Flavonifractor plautii*, showed the most favorable docking score with *CYP1B1*, suggesting a potential interaction. This predicted interaction warrants experimental evaluation because its effect on *CYP1B1* activity cannot be inferred from docking alone. Previous studies have also confirmed that HMCA possesses antidiabetic properties, anticancer activity, and cognitive-enhancing effects [32]. However, the specific associations among HMCA, *CYP1B1*, metabolic pathways, and the comorbidity of T2DM and ACI remain unclear.

Based on these findings, we hypothesize that the gut microbiota-derived metabolite HMCA may interact with *CYP1B1* and potentially alter its enzymatic activity. Because *CYP1B1* participates in tryptophan metabolism and steroid hormone biosynthesis, this interaction could be associated with changes in indole derivatives, estradiol metabolism, or AHR-related signaling. However, molecular docking alone cannot establish *CYP1B1* inhibition or downstream effects on insulin resistance and post-stroke inflammation; these links require enzymatic assays, targeted metabolomics, and disease-model validation.

*ANPEP* participates in protein processing, amino acid metabolism and cholesterol uptake, and broadly governs multiple vital physiological processes including angiogenesis and immune responses [33]. Accumulating evidence demonstrates that *ANPEP* is markedly upregulated in pancreatic β-cells from patients with T2DM, and it participates in T2DM pathogenesis by modulating islet function [34]. In addition, cigarette smoking elevates *ANPEP* expression by activating inflammatory and immune cascades, destabilizing atherosclerotic plaques, and consequently increasing the risk of ACI [35]. Functional enrichment analysis further indicated that *ANPEP* was associated with the renin–angiotensin system and Glutathione metabolism pathways. The renin–angiotensin system is indispensable for the initiation and progression of cardiovascular, renal and metabolic disorders [36]. *ANPEP* is a key metabolic enzyme in the RAS that hydrolyzes angiotensin III to angiotensin IV, which in turn inhibits sodium reabsorption in the proximal renal tubules, increases renal cortical blood flow, and lowers blood pressure [37]. Hypertension and cerebral hypoperfusion are key drivers of disease progression in T2DM complicated by ACI. Thus, *ANPEP* may influence blood-pressure- and perfusion-related processes; however, its net effect in T2DM complicated by ACI remains uncertain. The glutathione (GSH) metabolic pathway is intimately linked to both T2DM and ACI. As the primary intracellular reducing agent and a potent antioxidant, GSH protects cells from oxidative damage. Its depletion leads to excessive ROS production and amplified inflammatory responses. Endogenous GSH deficiency is recognized as a key pathological trigger of oxidative stress, pancreatic β-cell apoptosis, and ultimately T2DM development [38]. Concurrently, GSH deficiency induces oxidative stress and apoptosis in vascular endothelial cells, thereby exacerbating cerebral infarction and post-infarction cerebral edema [39]. *ANPEP* contributes to GSH metabolism by generating cysteine and glycine for de novo GSH biosynthesis [40]. Korvyakova et al. [38] proposed that elevated *ANPEP* levels in T2DM patients reflect an adaptive response aimed at counteracting GSH deficiency. By generating GSH precursors, *ANPEP* helps restore glutathione balance, thereby maintaining cellular redox homeostasis and mitigating pancreatic β-cell damage and apoptosis. The updated KEGG analysis also highlighted Phagocytosis and Antigen processing and presentation, which are consistent with the lysosomal protease function of *CTSS*. In addition, Ovarian steroidogenesis and Chemical carcinogenesis-DNA adducts were enriched, further supporting a role for *CYP1B1*-mediated steroid and xenobiotic metabolism in the comorbidity. To date, how *ANPEP* influences ACI onset and progression via the GSH metabolic pathway remains unexplored. Collectively, *ANPEP* may be associated with T2DM-related redox dysregulation, whereas its role in ACI and the direction of its effects require direct validation.

This study integrated 16S rRNA sequencing and random forest analysis and identified *Bifidobacterium* as a pivotal microbial taxon that differentiates patients with T2DM complicated by ACI from those with isolated ACI. In laying-hen models, Xu et al. [41] demonstrated that dietary curcumin supplementation simultaneously upregulates intestinal *ANPEP* mRNA expression and elevates the relative abundance of genera including *Bifidobacterium* and *Alistipes*. Although their research did not explore the interplay between *ANPEP* and these microbes, the concurrent upregulation of *ANPEP* and enrichment of *Bifidobacterium* induced by curcumin suggests a possible association but does not establish a shared mechanism in humans. *Bifidobacterium* participates centrally in methionine metabolism [42], and methionine can mitigate systemic inflammation [43] and improve insulin sensitivity [44]. Our molecular docking results indicated that methionine, a metabolite derived from *Bifidobacterium*, showed a predicted interaction with *ANPEP*; however, docking cannot determine whether methionine acts as a substrate, activator, or inhibitor. Therefore, the co-occurrence of *Bifidobacterium* abundance and *ANPEP*-related signals may represent a compensatory association rather than a confirmed protective mechanism. An increased relative abundance of *Bifidobacterium* has been associated with intestinal homeostasis and inflammatory modulation [45,46]; however, whether microbially associated methionine alters *ANPEP* activity remains unknown. *ANPEP*-related effects on GSH homeostasis, endothelial integrity, neuronal oxidative injury, and inflammatory infiltration are therefore hypothetical in this comorbidity context. The proposed *Bifidobacterium*–methionine–*ANPEP* axis should be regarded as a testable model rather than a confirmed protective cascade. Moreover, the directionally inconsistent associations for *Alistipes* and *Bifidobacterium* further indicate that their roles may be context-dependent and require experimental validation.

Database-based prediction revealed that five TFs, namely SPI1, STAT3, CEBPB, ESR1, and FOXA1, were predicted as shared upstream regulators of *ANPEP* and *CYP1B1*, suggesting a potentially convergent regulatory layer rather than demonstrated synergistic effects. STAT3 has been implicated in metabolic regulation in T2DM. Existing studies demonstrate that STAT3 pathway activation suppresses fatty acid synthesis and deposition in pancreatic cells from high-fat diet-induced diabetic mice, thus protecting pancreatic β-cells from injury [47]. STAT3 represents a novel regulator of mitochondrial gene expression in pancreatic β-cells in obesity [48]. In addition, the JAK2/STAT3 cascade drives microglia/macrophage polarization toward the anti-inflammatory M2 phenotype, which mitigates cerebral ischemia–reperfusion injury [49]. ESR1 encodes estrogen receptor α, and ESR1 loss elevates the risks of obesity, tissue fibrosis and insulin resistance [50]. These findings provide biological context for STAT3 and ESR1 but do not establish their regulation of *ANPEP* or *CYP1B1* in T2DM complicated by ACI; the predicted relationships require experimental validation.

This study further predicted candidate compounds targeting *ANPEP* and *CYP1B1*. According to the results, chrysin was linked to *ANPEP*, whereas colchicine was linked to *CYP1B1*. Chrysin is a natural flavonoid with anti-inflammatory and antioxidant properties and has been investigated in metabolic disorders [51]. In athymic nude diabetic mice, chrysin reduced blood glucose and triglyceride levels and alleviated systemic inflammation, supporting its broader antidiabetic potential [52]. Colchicine, a natural alkaloid derived from Colchicum species, is the first anti-inflammatory agent approved by the U.S. Food and Drug Administration (FDA) for the treatment of coronary heart disease [53]. Wang et al. [54] provided evidence that colchicine can suppress inflammatory cascades and improve plaque-related outcomes in a diabetic atherosclerosis model. These findings support the general anti-inflammatory plausibility of the predicted compounds but do not validate the specific drug–gene interactions or efficacy in T2DM complicated by ACI. Therefore, the nominated pairs should be considered exploratory and require target-engagement, dose–response, and in vivo validation.

This study identified *ANPEP* and *CYP1B1* as candidate molecular features associated with gut microbial metabolites in T2DM complicated by ACI. However, several limitations should be acknowledged. First, the clinical 16S cohort was small and included only ACI and T2DM-ACI groups, without healthy or T2DM-only controls; residual confounding arising from diet, laxatives, iron supplements and other microbiota-altering medications cannot be fully excluded, although participants taking antibiotics, antiplatelet therapy, metformin, and acarbose were excluded. Although all participants were Han Chinese from the Kunming region, which reduces regional and ethnic heterogeneity, dietary habits were not systematically recorded. These factors may limit generalizability and attribution of microbial changes specifically to the comorbidity. Second, the GM and disease GWAS datasets were predominantly derived from European populations, whereas the clinical sequencing cohort was recruited at a Chinese center. Cross-tissue and cross-ancestry integration may limit direct comparability and generalizability. Third, the use of a non-T2DM-specific GWAS endpoint may introduce phenotypic heterogeneity; the MR findings should therefore be interpreted as supporting T2DM-related genetic susceptibility rather than definitive T2DM-specific causality. Fourth, metabolite–target prediction, regulatory-network analysis, and molecular docking provide hypothesis-generating evidence but do not confirm metabolite abundance, target engagement, interaction direction, or biological function. Fifth, multiple testing correction was not uniformly applied across all exploratory analyses. MR causal screening and differential expression analysis used nominal *p* < 0.05 thresholds without FDR or Bonferroni correction, whereas GO and KEGG enrichment results were reported using BH-FDR-adjusted *p*.adjust < 0.05. This inconsistency may increase the risk of false-positive findings in the exploratory steps, and the MR and DEG results should therefore be interpreted as hypothesis-generating rather than definitive. Future studies should integrate multicenter, multi-ethnic cohorts with targeted metabolomics, enzymatic and gene-perturbation assays, and appropriate animal models.

## 5. Conclusions

In summary, this study combined bioinformatic analysis and 16S rRNA sequencing to identify core genes and key gut microbial taxa as candidate molecular features for T2DM-related genetic susceptibility and ACI, and further characterized their diverse biological functions and signaling pathways associated with disease progression. The present work establishes a systematic bioinformatic foundation for deciphering the molecular mechanisms associated with T2DM complicated by ACI. Nevertheless, additional experimental research is still required to validate the proposed regulatory relationships in disease initiation and progression. The predicted *ANPEP*/*CYP1B1*-related networks and candidate drugs require enzymatic assays, targeted metabolomics and in vivo validation. These findings should be interpreted as supporting T2DM-related genetic susceptibility rather than strict T2DM-specific causality.

## Figures and Tables

**Figure 1:**
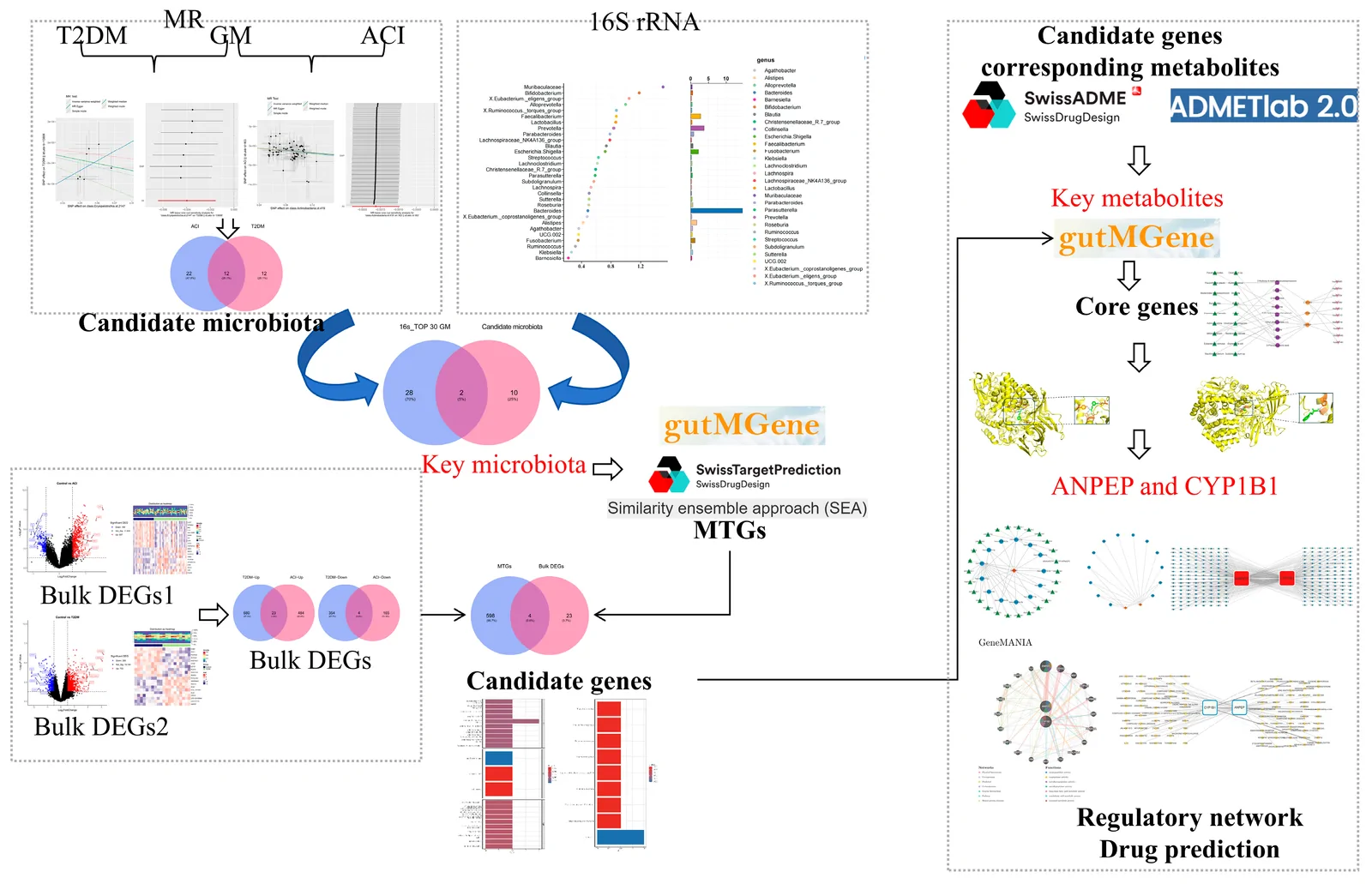
Flow diagram of the study.

**Figure 2:**
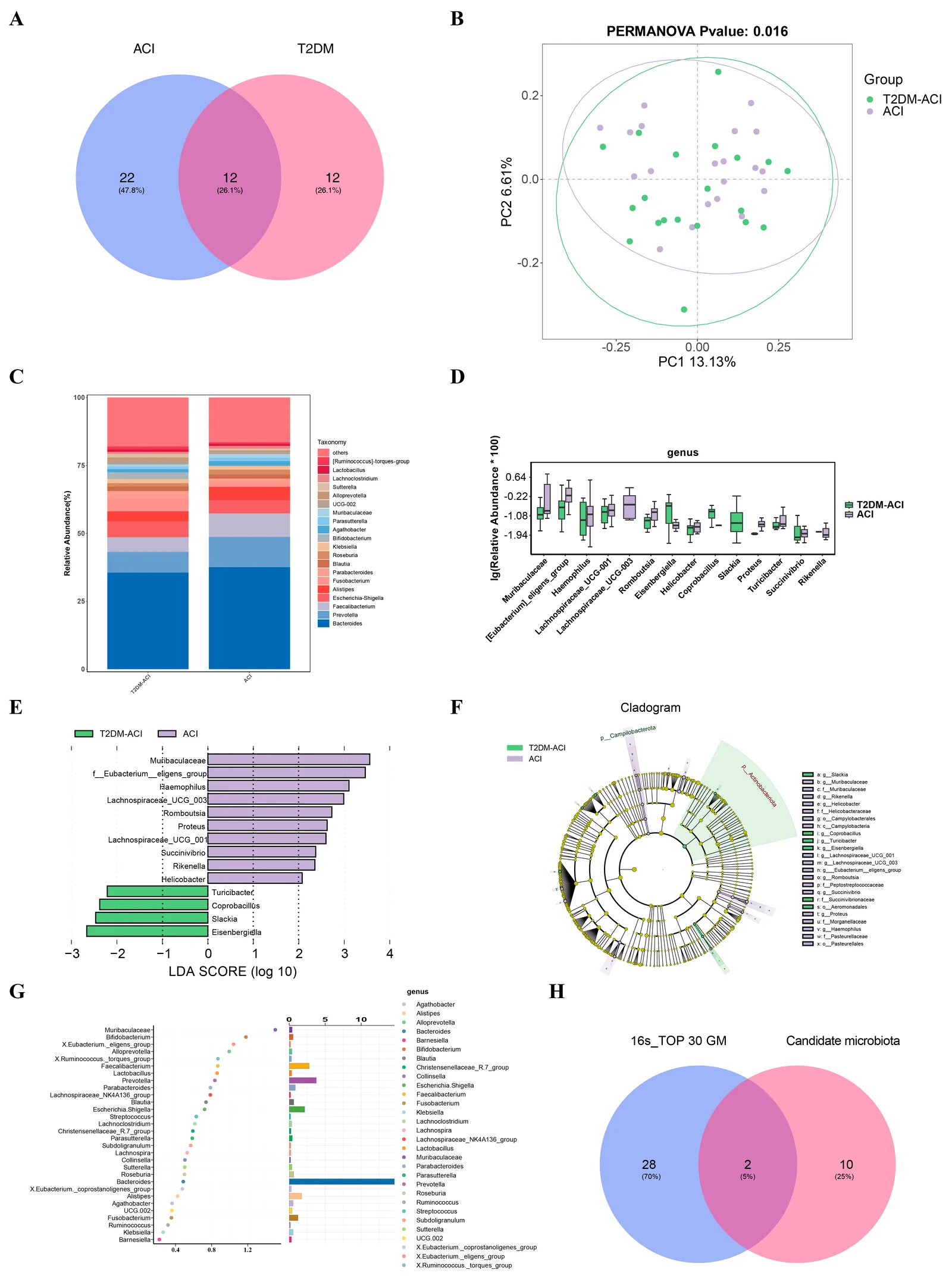
Screening of key microbial taxa. (**A**) Venn diagram showing the intersection of 24 T2DM-associated gut microbiota (GM) and 34 ACI-associated GM. (**B**) PCoA based on unweighted UniFrac distance(Each dot represents one sample. Each colored ellipse represents the 95% confidence ellipse for the corresponding group.). (**C**) Relative abundance analysis at the genus level (top 20 in relative abundance). (**D**) Box plots of differential taxa at the genus level (top 20 in relative abundance). (**E**) Column diagram of LDA for LEfSe at the genus level. (**F**) Cladogram of LDA for LEfSe at the genus level. (**G**) Dot plot of important taxa based on random forest analysis at the genus level. (**H**) Venn diagram showing the intersection of 12 candidate microbial taxa and the top 30 taxa from 16S rRNA sequencing.

**Figure 3:**
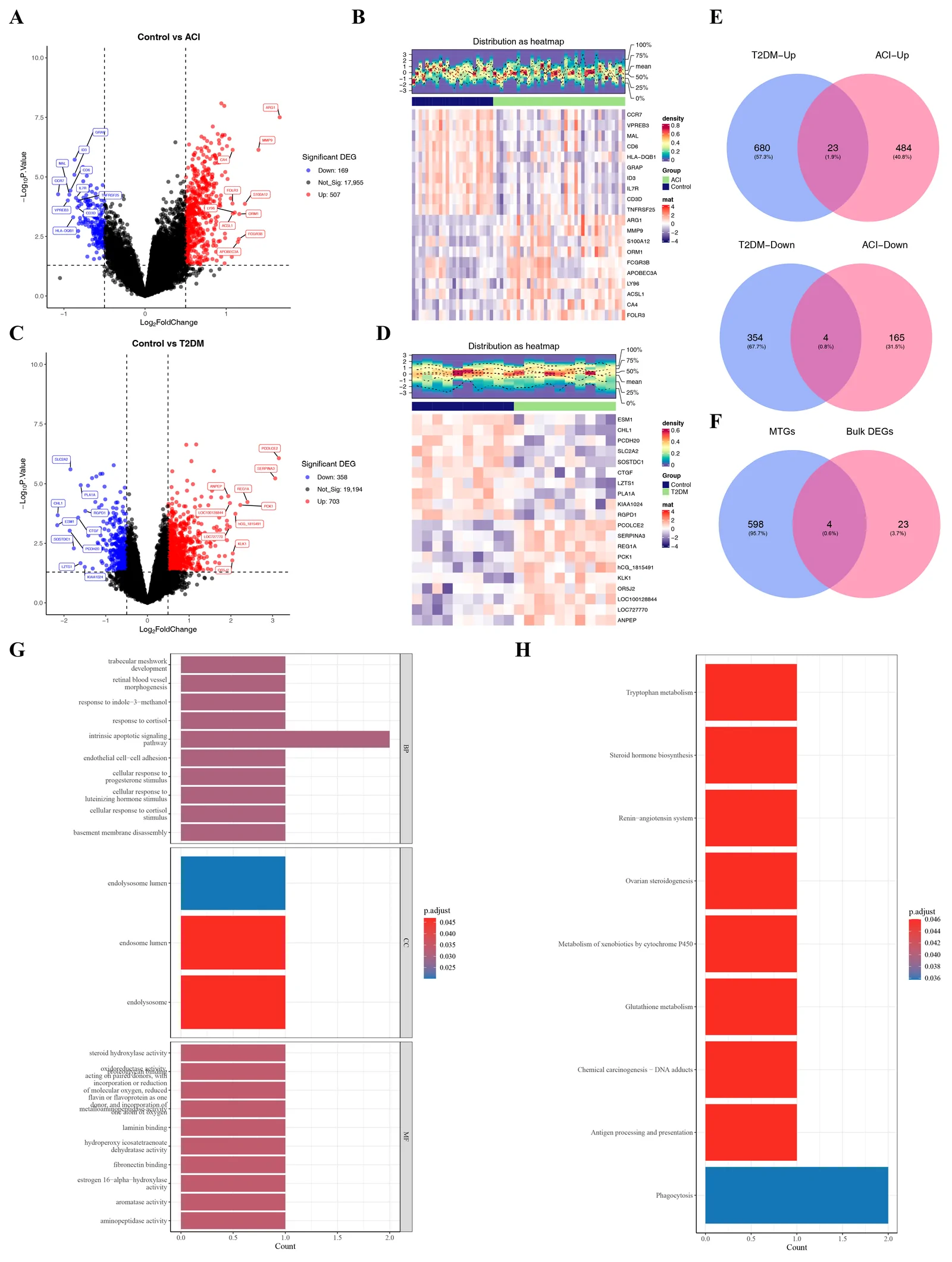
Screening of candidate genes. (**A**) Volcano plot of 676 bulk differentially expressed genes(DEGs) between ACIand control samples in dataset GSE16561. The horizontal reference line indicates −log_10_(0.05)=1.3, and the vertical reference lines denote log_2_FC = ±0.5. (**B**) Heatmap of 676 bulk DEGs between ACIand control samples in dataset GSE16561. (**C**) Volcano plot of 1061 bulk DEGs between T2DMand control samples in dataset GSE20966. (**D**) Heatmap of 1061 bulk DEGs between T2DMand control samples in dataset GSE20966. (**E**) Venn diagram showing the intersection of upregulated/downregulated genes between 1061 bulk DEGs and 676 bulk DEGs. (**F**) Venn diagram showing the intersection of 27 bulk DEGs and 602 metabolite–target genes (MTGs). (**G**) Gene Ontology (GO) enrichment analyses of candidate genes. (**H**) Kyoto Encyclopedia of Genes and Genomes (KEGG) enrichment analyses of candidate genes.

**Figure 4:**
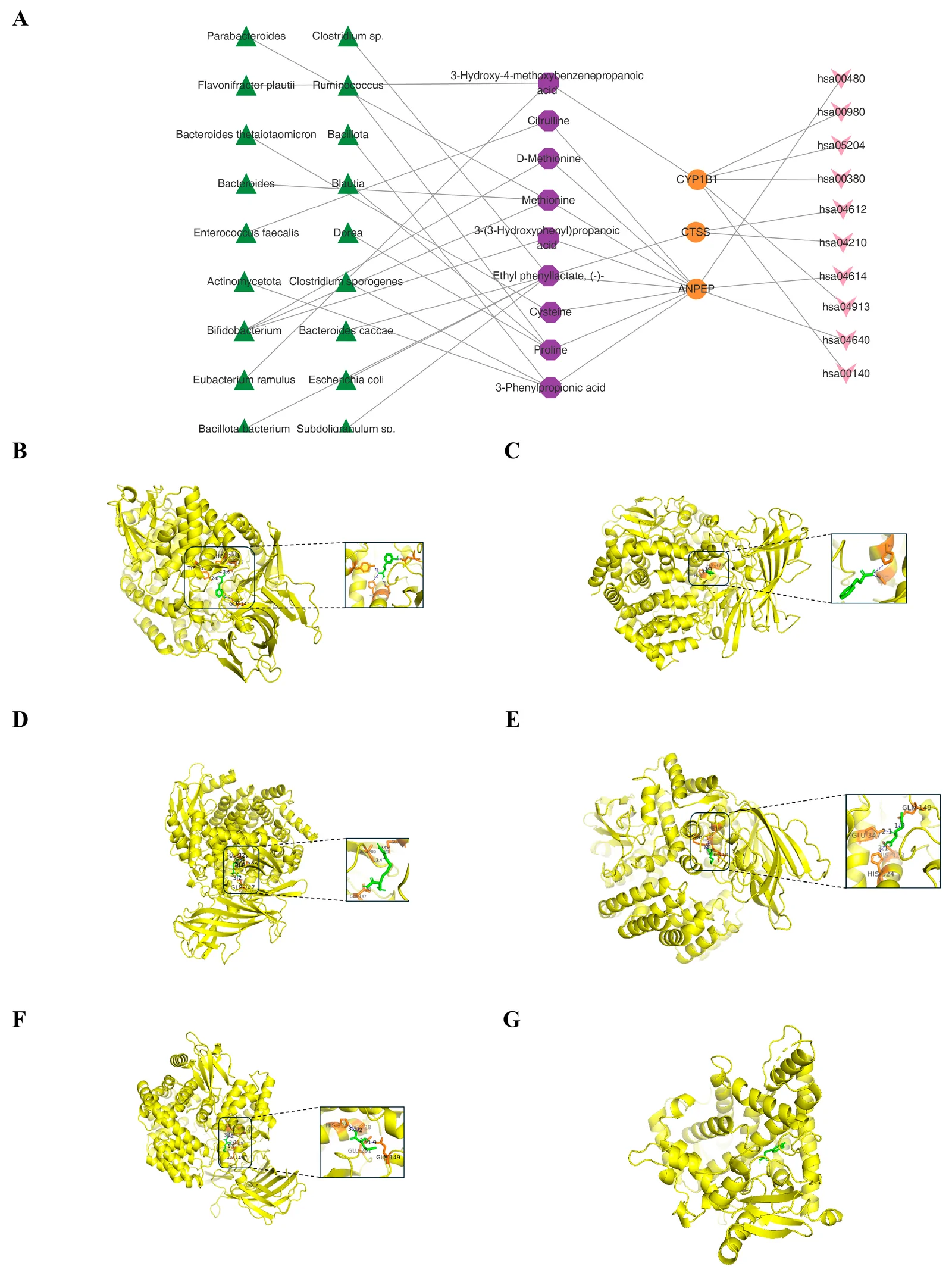
Identification of candidate key genes. (**A**) Regulatory network of 3 core genes, 18 GMs, 9 key metabolites, and 9 KEGG pathways. (**B**) Predicted molecular docking results of *ANPEP* with 3-(3-Hydroxyphenyl)propanoic acid. (**C**) Predicted molecular docking results of *ANPEP* with 3-phenylpropionic acid. (**D**) Predicted molecular docking results of *ANPEP* with citrulline. (**E**) Predicted molecular docking results of *ANPEP* with D-methionine. (**F**) Predicted molecular docking results of *ANPEP* with methionine. (**G**) Predicted molecular docking results of *CYP1B1* with 3-hydroxy-4-methoxybenzenepropanoic acid.

**Figure 5:**
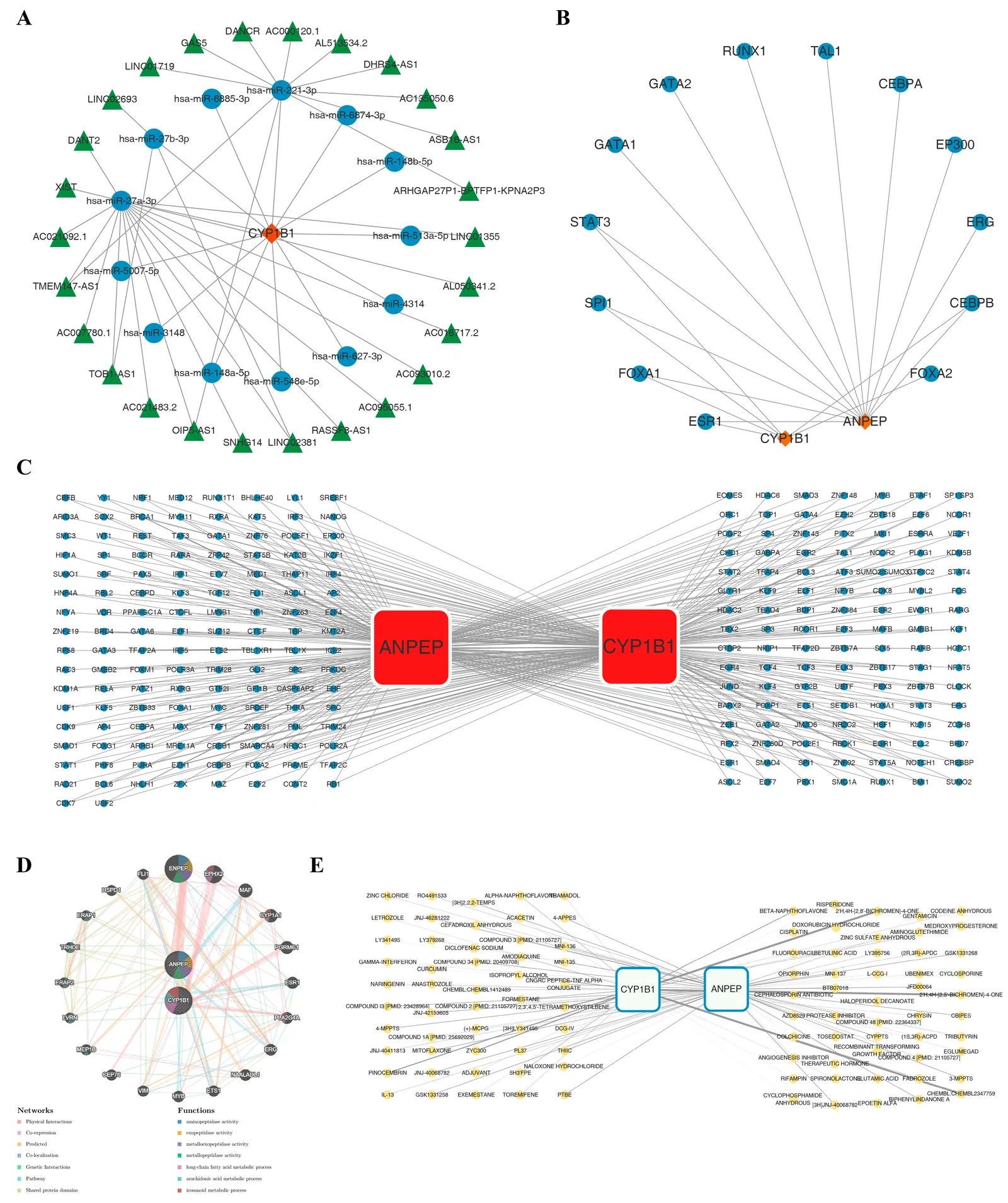
Regulatory network and drug prediction analysis of candidate key genes. (**A**) Long noncoding RNAs (lncRNAs)–microRNAs–mRNA regulatory network. In this figure, different colors distinguish distinct biological components: red nodes represent candidate key genes, blue nodes denote miRNAs, and green nodes stand for lncRNAs. (**B**) mRNA–transcription factor (TF) network. In this figure, different colors are used to distinguish distinct biological components: orange nodes represent candidate key genes and blue nodes denote TFs. (**C**) TF regulatory network based on transcription factor binding site (TFBS) prediction. (**D**) GeneMANIA analysis of candidate key genes. (**E**) Interaction network between candidate key genes and targeted drugs. White nodes represent candidate key genes, and yellow nodes denote predicted drugs.

**Table 1:** Core genes and key gut microbial metabolites.

Gene	Metabolite	Compound_SMILES_swissadme	Medicine	Compound_SMILES_ADMETlab	Toxicity
*ANPEP*	3-(3-Hydroxyphenyl)propanoic acid	OC(=O)CCc1cccc(c1)O	TRUE	O=C(O)CCc1cccc(O)c1	FALSE
*ANPEP*	3-Phenylpropionic acid	OC(=O)CCc1ccccc1	TRUE	O=C(O)CCc1ccccc1	FALSE
*ANPEP*	Citrulline	NC(=O)NCCC[C@@H](C(=O)O)N	TRUE	NC(=O)NCCCC(N)C(=O)O	FALSE
*ANPEP*	Cysteine	N[C@H](C(=O)O)CS	TRUE	NC(CS)C(=O)O	FALSE
*ANPEP*	D-Methionine	CSCC[C@H](C(=O)O)N	TRUE	CSCCC(N)C(=O)O	FALSE
*ANPEP*	Methionine	CSCC[C@@H](C(=O)O)N	TRUE	CSCCC(N)C(=O)O	FALSE
*ANPEP*	Proline	OC(=O)[C@@H]1CCCN1	TRUE	O=C(O)C1CCCN1	FALSE
*CYP1B1*	3-Hydroxy-4-methoxybenzenepropanoic acid	COc1ccc(cc1O)CCC(=O)O	TRUE	COc1ccc(CCC(=O)O)cc1O	FALSE
*CTSS*	Ethyl phenyllactate, (-)-	CCOC(=O)[C@H](Cc1ccccc1)O	TRUE	CCOC(=O)C(O)Cc1ccccc1	FALSE

**Table 2:** Molecular docking score summary.

Target	Metabolite	Binding Energy (kcal/mol)
*ANPEP*	3-(3-Hydroxyphenyl)propanoic acid	−4.3
	3-Phenylpropionic acid	−5.1
	Citrulline	−4.3
	Cysteine	−4.1
	D-Methionine	−4.5
	Methionine	−4.5
	Proline	−3.9
*CTSS*	Ethyl phenyllactate, (-)-	−2.9
*CYP1B1*	3-Hydroxy-4-methoxybenzenepropanoic acid	−4.5

## Data Availability

Publicly available datasets were analyzed in this study. The gene-expression datasets GSE20966 (platform GPL1352) and GSE16561 (platform GPL6883) were downloaded from the Gene Expression Omnibus (GEO) database (https://www.ncbi.nlm.nih.gov/geo/, accessed on 13 October 2025). GWAS summary statistics for type 2 diabetes mellitus (GWAS ID: finn-b-E4_DIABETES) and cerebral infarction (GWAS ID: ukb-d-I63) were obtained from the IEU OpenGWAS database (https://gwas.mrcieu.ac.uk/, accessed on 14 October 2025). GWAS summary data related to gut microbiota were obtained from the MiBioGen Consortium (www.mibiogen.org, accessed on 14 October 2025). Other data supporting the findings of this study are available from the corresponding author upon request.

## Supplementary Materials

The following supporting information can be downloaded at https://www.mdpi.com/article/10.3390/biomedicines14102371/s1: Figure S1: Scatter plots illustrating associations of 24 gut microbiota taxa with T2DM risk and 34 gut microbiota taxa with ACI risk; Figure S2: Analysis of diagnostic efficacy of gut microbiota for T2DM and ACI; Figure S3: Random-effects estimation for single-nucleotide polymorphisms (SNPs) of gut microbiota in T2DM and ACI; Figure S4: Leave-one-out validation for gut microbiota in T2DM and ACI; Figure S5: Box plots of differences in alpha diversity indices; Table S1: List of key microbiota-related metabolites and MTGs; Table S2: Instrumental variable (IV) selection for MR analysis of GM and T2DM/ACI; Table S3: Screening of 24 T2DM-associated and 34 ACI-associated candidate exposure factors; Table S4: Heterogeneity test results (Q test) for 24 T2DM-associated and 34 ACI-associated candidate exposure factors; Table S5: Horizontal pleiotropy test results for 24 T2DM-associated and 34 ACI-associated candidate exposure factors; Table S6: Steiger directionality analysis results for 24 candidate exposure factors and T2DM; Steiger directionality analysis results for 34 candidate exposure factors and ACI; Table S7: List of 43 key microbiota-related metabolites; Table S8: Differential expression analysis results; Table S9: GO and KEGG enrichment analysis results of candidate genes; Table S10: Druggability and toxicity evaluation results of candidate gene-interacting metabolites.

## Abbreviations

The following abbreviations are used in this manuscript:

**Abbreviation**	**Full Term**	**Abbreviation**	**Full Term**
ACI	acute cerebral infarction	LD	linkage disequilibrium
ADMET	absorption, distribution, metabolism, excretion, and toxicity	LEfSe	linear discriminant analysis effect size
AHR	aryl hydrocarbon receptor	lncRNA	long noncoding RNA
*ANPEP*	alanyl aminopeptidase	MR	Mendelian randomization
AS	atherosclerosis	MTG	metabolite–target gene
ASV	amplicon sequence variant	NCBI	National Center for Biotechnology Information
BP	biological process	OR	odds ratio
CC	cellular component	PCoA	principal coordinates analysis
*CYP1B1*	cytochrome P450 family 1 subfamily B member 1	PDB	Protein Data Bank
DEG	differentially expressed gene	PERMANOVA	permutational multivariate analysis of variance
DGIdb	Drug–Gene Interaction Database	PPAR-α	peroxisome proliferator-activated receptor alpha
DILI	drug-induced liver injury	RCSB	Research Collaboratory for Structural Bioinformatics
FDA	U.S. Food and Drug Administration	RF	random forest
GM	gut microbiota	ROS	reactive oxygen species
GO	Gene Ontology	SEA	Similarity Ensemble Approach
GSH	glutathione	SMILES	simplified molecular-input line-entry system
GWAS	genome-wide association study	SNP	single-nucleotide polymorphism
HBA	hydrogen-bond acceptor	STP	SwissTargetPrediction
HBD	hydrogen-bond donor	T2DM	type 2 diabetes mellitus
HMCA	3-hydroxy-4-methoxybenzenepropanoic acid	TF	transcription factor
IV	instrumental variable	TFBS	transcription factor binding site
IVW	inverse-variance weighted	TPSA	topological polar surface area
KEGG	Kyoto Encyclopedia of Genes and Genomes	FDR	false discovery rate

## References

[1] Genitsaridi I., Salpea P., Salim A., Sajjadi S.F., Tomic D., James S., Thirunavukkarasu S., Issaka A., Chen L., Basit A. (2026). 11th edition of the IDF Diabetes Atlas: Global, regional, and national diabetes prevalence estimates for 2024 and projections for 2050. Lancet Diabetes Endocrinol..

[2] Mosenzon O., Cheng A.Y., Rabinstein A.A., Sacco S. (2023). Diabetes and Stroke: What Are the Connections?. J. Stroke.

[3] Wang W., Jiang B., Sun H., Ru X., Sun D., Wang L., Wang L., Jiang Y., Li Y., Wang Y. (2017). Prevalence, Incidence, and Mortality of Stroke in China: Results from a Nationwide Population-Based Survey of 480 687 Adults. Circulation.

[4] Edwards M.D., Hughes T.A.T. (2021). Managing blood pressure in acute cerebral infarction. J. Neurol..

[5] Kristensen S.L., Lindhardsen J., Ahlehoff O., Erichsen R., Lamberts M., Khalid U., Torp-Pedersen C., Nielsen O.H., Gislason G.H., Hansen P.R. (2014). Increased risk of atrial fibrillation and stroke during active stages of inflammatory bowel disease: A nationwide study. Europace.

[6] Wang H., Song W., Wu Q., Gao X., Li J., Tan C., Zhou H., Zhu J., He Y., Yin J. (2021). Fecal Transplantation from db/db Mice Treated with Sodium Butyrate Attenuates Ischemic Stroke Injury. Microbiol. Spectr..

[7] Zhang J., Wang T., Yang P., Miao Y., Ge B., Sun J. (2025). Association of Sleep Traits, Physical Activity, and Sedentary Leisure Behavior With Female Reproductive Health: A Two-Sample Mendelian Randomization Analysis. Int. J. Womens Health.

[8] Meng C., Deng P., Miao R., Tang H., Li Y., Wang J., Wu J., Wang W., Liu S., Xia J. (2023). Gut microbiome and risk of ischaemic stroke: A comprehensive Mendelian randomization study. Eur. J. Prev. Cardiol..

[9] Bao X., Zeng Z., Tang W., Li D., Fan X., Chen K., Wang Y., Ai W., Yang Q., Liu S. (2025). Bioinformatics Combined With Biological Experiments to Identify the Pathogenetic Link of Type 2 Diabetes for Breast Cancer. Cancer Med..

[10] Chen W., Chen Y., Wu L., Gao Y., Zhu H., Li Y., Ji X., Wang Z., Wang W., Han L. (2023). Identification of cell death-related biomarkers and immune infiltration in ischemic stroke between male and female patients. Front. Immunol..

[11] Kurilshikov A., Medina-Gomez C., Bacigalupe R., Radjabzadeh D., Wang J., Demirkan A., Le Roy C.I., Raygoza Garay J.A., Finnicum C.T., Liu X. (2021). Large-scale association analyses identify host factors influencing human gut microbiome composition. Nat. Genet..

[12] Hemani G., Zheng J., Elsworth B., Wade K.H., Haberland V., Baird D., Laurin C., Burgess S., Bowden J., Langdon R. (2018). The MR-Base platform supports systematic causal inference across the human phenome. Elife.

[13] American Diabetes Association Professional Practice Committee for Diabetes (2024). 2. Diagnosis and Classification of Diabetes: Standards of Care in Diabetes-2024. Diabetes Care.

[14] Liu L., Li Z., Zhou H., Duan W., Huo X., Xu W., Li S., Nie X., Liu H., Liu J. (2023). Chinese Stroke Association guidelines for clinical management of ischaemic cerebrovascular diseases: Executive summary and 2023 update. Stroke Vasc. Neurol..

[15] Chen H., Boutros P.C. (2011). VennDiagram: A package for the generation of highly-customizable Venn and Euler diagrams in R. BMC Bioinform..

[16] Ritchie M.E., Phipson B., Wu D., Hu Y., Law C.W., Shi W., Smyth G.K. (2015). limma powers differential expression analyses for RNA-sequencing and microarray studies. Nucleic Acids Res..

[17] Gustavsson E.K., Zhang D., Reynolds R.H., Garcia-Ruiz S., Ryten M. (2022). ggtranscript: An R package for the visualization and interpretation of transcript isoforms using ggplot2. Bioinformatics.

[18] Li J., Wang H., Cao F., Cheng Y. (2022). A bioinformatics analysis for diagnostic roles of the E2F family in esophageal cancer. J. Gastrointest. Oncol..

[19] Yu G., Wang L.G., Han Y., He Q.Y. (2012). clusterProfiler: An R package for comparing biological themes among gene clusters. Omics.

[20] Shannon P., Markiel A., Ozier O., Baliga N.S., Wang J.T., Ramage D., Amin N., Schwikowski B., Ideker T. (2003). Cytoscape: A software environment for integrated models of biomolecular interaction networks. Genome Res..

[21] Ying K., Chen J., Fu Z., Ren B. (2023). FAS-mediated circRNA-miRNA-mRNA Crosstalk Network Regulates Immune Cell Infiltration in Cerebral Infarction. J. Mol. Neurosci..

[22] Zhang L., Chen Y., Xu J., Corpe C.P., Shtaya A., Benjamin P., Xu Y. (2024). Inflammation in ischemic stroke patients with type 2 diabetes—Part II: Potential therapeutic targets. Adv. Neurol..

[23] Holman R.R., Paul S.K., Bethel M.A., Matthews D.R., Neil H.A. (2008). 10-year follow-up of intensive glucose control in type 2 diabetes. N. Engl. J. Med..

[24] Chen C., Yang Y., Guo Y., He J., Chen Z., Qiu S., Zhang Y., Ding H., Pan J., Pan Y. (2023). *CYP1B1* inhibits ferroptosis and induces anti-PD-1 resistance by degrading ACSL4 in colorectal cancer. Cell Death Dis..

[25] Ononamadu C.J., Seidel V. (2024). Exploring the Antidiabetic Potential of Salvia officinalis Using Network Pharmacology, Molecular Docking and ADME/Drug-Likeness Predictions. Plants.

[26] Noreen Z., Loffredo C.A., Bhatti A., Simhadri J.J., Nunlee-Bland G., Nnanabu T., John P., Khan J.S., Ghosh S. (2020). Transcriptional Profiling and Biological Pathway(s) Analysis of Type 2 Diabetes Mellitus in a Pakistani Population. Int. J. Environ. Res. Public Health.

[27] Du Q., Liu K., Li Y., Wang X., Liu X., Zhao J., Wang X. (2025). Inhibition of CYP450 family 1 subfamily B member 1 (*CYP1B1*) expression in macrophage reduces the inflammatory response in type 2 diabetes mellitus combined with tuberculosis. Front. Endocrinol..

[28] Zhang B., Subramanian S., Dziennis S., Jia J., Uchida M., Akiyoshi K., Migliati E., Lewis A.D., Vandenbark A.A., Offner H. (2010). Estradiol and G1 reduce infarct size and improve immunosuppression after experimental stroke. J. Immunol..

[29] Tofovic S.P. (2010). Estrogens and development of pulmonary hypertension: Interaction of estradiol metabolism and pulmonary vascular disease. J. Cardiovasc. Pharmacol..

[30] Wang J.J., Xu F.B., Hu S., Xu Y.M., Wang X.Z. (2024). Identification and validation of cortisol-related hub biomarkers and the related pathogenesis of biomarkers in Ischemic Stroke. Brain Behav..

[31] Chen L.H., Lin W.R., Hu H.R., Chen Z.Y., Wang Y.H. (2025). Identification and validation of the important role of tryptophan-related gene *CYP1B1* in the development and progression of sepsis. PLoS ONE.

[32] Gao J., Mao K., Wang X., Mi S., Fu M., Li X., Xiao J., Simal-Gandara J., Sang Y. (2021). Tibet Kefir Milk Regulated Metabolic Changes Induced by High-Fat Diet via Amino Acids, Bile Acids, and Equol Metabolism in Human-Microbiota-Associated Rats. J. Agric. Food Chem..

[33] Ding N., Wang R., Wang P., Wang F. (2024). Metabolism-related proteins as biomarkers for predicting prognosis in polycystic ovary syndrome. Proteome Sci..

[34] Liang Y.C., Li L., Liang J.L., Liu D.L., Chu S.F., Li H.L. (2024). Integrating Mendelian randomization and single-cell RNA sequencing to identify therapeutic targets of baicalin for type 2 diabetes mellitus. Front. Pharmacol..

[35] Zhou S., Liu S., Liu X., Zhuang W. (2021). Bioinformatics Gene Analysis of Potential Biomarkers and Therapeutic Targets for Unstable Atherosclerotic Plaque-Related Stroke. J. Mol. Neurosci..

[36] Ichikawa M., Konoshita T., Makino Y., Suzuki J., Ishizuka T., Nakamura H. (2020). An association study of C9orf3, a novel component of the renin-angiotensin system, and hypertension in diabetes. Sci. Rep..

[37] Kotlo K., Hughes D.E., Herrera V.L., Ruiz-Opazo N., Costa R.H., Robey R.B., Danziger R.S. (2007). Functional polymorphism of the *Anpep* gene increases promoter activity in the Dahl salt-resistant rat. Hypertension.

[38] Korvyakova Y., Azarova I., Klyosova E., Postnikova M., Makarenko V., Bushueva O., Solodilova M., Polonikov A. (2025). The link between the *ANPEP* gene and type 2 diabetes mellitus may be mediated by the disruption of glutathione metabolism and redox homeostasis. Gene.

[39] Lan X.Y., Sun Z.W., Xu G.L., Chu C.Y., Qin H.M., Li S., Geng X., Gao P., Boltze J., Li S. (2022). Bone Marrow Mesenchymal Stem Cells Exert Protective Effects After Ischemic Stroke Through Upregulation of Glutathione. Stem Cell Rev. Rep..

[40] Klyosova E., Azarova I., Buikin S., Polonikov A. (2023). Differentially Expressed Genes Regulating Glutathione Metabolism, Protein-Folding, and Unfolded Protein Response in Pancreatic β-Cells in Type 2 Diabetes Mellitus. Int. J. Mol. Sci..

[41] Xu Z., Zhu W., Xu D., Amevor F.K., Wu Y., Ma D., Cao X., Wei S., Shu G., Zhao X. (2024). Supplementation of curcumin promotes the intestinal structure, immune barrier function and cecal microbiota composition of laying hens in early laying period. Poult. Sci..

[42] Kim Y.T., Kwon J.G., O’Sullivan D.J., Lee J.H. (2024). Regulatory mechanism of cysteine-dependent methionine biosynthesis in *Bifidobacterium* longum: Insights into sulfur metabolism in gut microbiota. Gut Microbes.

[43] Troha K., Kambhampati S., Insenga A., Metallo C.M., Ayres J.S. (2026). Dietary methionine mitigates immune-mediated damage by enhancing renal clearance of cytokines. Cell Metab..

[44] Li C., Gao L., Lv C., Li Z., Fan S., Liu X., Rong X., Huang Y., Liu J. (2023). Active role of amino acid metabolism in early diagnosis and treatment of diabetic kidney disease. Front. Nutr..

[45] Jang L.G., Choi G., Kim S.W., Kim B.Y., Lee S., Park H. (2019). The combination of sport and sport-specific diet is associated with characteristics of gut microbiota: An observational study. J. Int. Soc. Sports Nutr..

[46] Bao W., Sun Y., Wang J., Wei S., Mao L., Zheng J., Liu P., Yang X., Chen Z. (2024). Relationship Between the Gut Microbiota and Neurological Deficits in Patients With Cerebral Ischemic Stroke. Neurorehabil. Neural Repair.

[47] Tiano J.P., Delghingaro-Augusto V., Le May C., Liu S., Kaw M.K., Khuder S.S., Latour M.G., Bhatt S.A., Korach K.S., Najjar S.M. (2011). Estrogen receptor activation reduces lipid synthesis in pancreatic islets and prevents β cell failure in rodent models of type 2 diabetes. J. Clin. Investig..

[48] Schaschkow A., Pang L., Vandenbempt V., Elvira B., Litwak S.A., Vekeriotaite B., Maillard E., Vermeersch M., Paula F.M.M., Pinget M. (2021). STAT3 Regulates Mitochondrial Gene Expression in Pancreatic β-Cells and Its Deficiency Induces Glucose Intolerance in Obesity. Diabetes.

[49] Fu S., Tang W., Luo S., Wang W., Huang Y., Li H., Wang G. (2026). Identification of diagnostic biomarkers for ischemic stroke and drug targets of Buyang Huanwu decoction: A bioinformatics and network pharmacology study. Medicine.

[50] Fatima L.A., Campello R.S., Santos R.S., Freitas H.S., Frank A.P., Machado U.F., Clegg D.J. (2017). Estrogen receptor 1 (ESR1) regulates VEGFA in adipose tissue. Sci. Rep..

[51] Hosseini S.A., Ajorlou P., Mousavi P., Aghamirli S., Dehghani A. (2026). Molecular crosstalk between AHR, *CYP1B1*, and ITGAM in diabetic nephropathy: Integrated insights from bioinformatics and experimental validation. Biochem. Biophys. Rep..

[52] Ramírez-Espinosa J.J., Saldaña-Ríos J., García-Jiménez S., Villalobos-Molina R., Ávila-Villarreal G., Rodríguez-Ocampo A.N., Bernal-Fernández G., Estrada-Soto S. (2017). Chrysin Induces Antidiabetic, Antidyslipidemic and Anti-Inflammatory Effects in Athymic Nude Diabetic Mice. Molecules.

[53] Wang H., Jiang M., Li X., Zhao Y., Shao J., Liu Z., Lin L., Xu Q., Wang L., Lu X. (2021). Anti-inflammatory Therapies for Coronary Heart Disease: A Systematic Review and Meta-Analysis. Front. Cardiovasc. Med..

[54] Wang H., Feng W., Wang J., Zhang Y., Han F., Hou Y., Hu S., Li B., Mu Y., Zhang R. (2025). Colchicine alleviates atherosclerosis combined with diabetes mellitus by targeting PIM2 and regulating the NF-κB signaling pathway. Phytomedicine.

